# Behandlung von Menschen, die wegen einer Sexualstraftat inhaftiert sind: Ansätze und empirische Befunde

**DOI:** 10.1007/s00103-025-04152-z

**Published:** 2025-11-04

**Authors:** Isabella Krupp, Fritjof von Franqué, Peer Briken, Alexander Voulgaris

**Affiliations:** Institut für Sexualforschung, Sexualmedizin und Forensische Psychiatrie (W38), Universitätsklinikum Hamburg-Eppendorf, Martinistraße 52, 20249 Hamburg, Deutschland

**Keywords:** Sexualstraftaten, Strafvollzug, Behandlung, Evaluation, Behandlungseffekte, Sexual offending, Prison, Treatment, Evaluation, Treatment efficacy

## Abstract

Sexualdelikte verursachen bedeutende individuelle und gesellschaftliche Schäden. Psychosoziale und psychotherapeutische Interventionen gelten dabei als zentrale Komponenten der Rückfallprävention. Die internationale Evidenz zur Wirksamkeit von Ansätzen zur Behandlung von Menschen, die wegen der Begehung einer Sexualstraftat inhaftiert sind, ist jedoch uneinheitlich und durch methodische Limitationen geprägt. Diese Übersichtsarbeit untersucht den aktuellen Forschungsstand in Deutschland. Zu diesem Zweck wurde die Literatur systematisch untersucht. Ziel ist es, die Evidenz zur Effektivität der Behandlungsansätze aus den letzten 25 Jahren zusammenzufassen und unter besonderer Berücksichtigung methodischer Aspekte zu beurteilen.

Empirische Studien deuten darauf hin, dass die Behandlung positive Effekte auf die allgemeine Legalbewährung (d. h. das strafrechtlich relevante Verhalten nach der Entlassung) haben kann. Jedoch gibt es keine klaren Hinweise auf eine Reduktion sexualbezogener Rückfälle. Behandlungsabbrüche gingen mit erhöhten Rückfallquoten einher. Untersuchungen zu Veränderungen risikorelevanter Merkmale berichten teils günstige Entwicklungen, doch die stark heterogene Qualität und Methodik der Studien begrenzen die Aussagekraft.

Insgesamt lässt sich derzeit kein abschließendes Urteil über die Wirksamkeit intramuraler Behandlungsangebote in Deutschland treffen. Es besteht ein deutlicher Bedarf an größeren, methodisch robusten Studien mit äquivalenten Vergleichsgruppen und detaillierter Erfassung der Behandlungsinhalte, um belastbare Evidenz zur Effektivität dieser Interventionen zu gewinnen.

## Abstract (en)

Sexual offenses cause significant individual and societal harm. Psychosocial and psychotherapeutic interventions are considered central components of relapse prevention. However, international evidence on the effectiveness of approaches for treating individuals incarcerated for sexual offenses is inconsistent and marked by methodological limitations. This review examines the current state of research in Germany. For this purpose, the literature was systematically reviewed. The aim is to summarize the evidence on the effectiveness of treatment approaches over the past 25 years and to assess it with particular attention to methodological aspects.

Empirical studies indicate that treatment can have positive effects on general recidivism (i.e., reoffending after release). However, there is no clear evidence for a reduction in sexual reoffending. Treatment dropout was associated with higher recidivism rates. Studies examining changes in risk-relevant factors report some favorable developments, but the highly heterogeneous quality and methodology of these studies limit the strength of the conclusions that can be drawn.

Overall, it is currently not possible to make a definitive judgment about the effectiveness of inpatient treatment programs for sexual offenders in Germany. There is a clear need for larger, methodologically robust studies with equivalent comparison groups and detailed documentation of treatment content in order to generate reliable evidence on the effectiveness of these interventions.

## Einleitung

Der Umgang mit Menschen, die ein Sexualdelikt verübt haben, stellt eine besonders herausfordernde gesellschaftliche Aufgabe dar. Sexualdelikte erhalten in der Öffentlichkeit große Aufmerksamkeit [1], obwohl aktuelle Studien im deutschsprachigen Raum verhältnismäßig niedrige einschlägige Rückfallraten berichten [2, 3]. Gleichwohl machen die erheblichen individuellen und sozialen Folgen solcher Taten die Notwendigkeit wirksamer Rückfallprävention deutlich. Psychosoziale Behandlungsangebote im Strafvollzug bilden dabei einen zentralen Bestandteil der Prävention.

Ob, in welchem Ausmaß und auf welche Weise intramurale, also im Strafvollzug stattfindende Behandlung zur Prävention von Sexualdelinquenz beiträgt, wird kontrovers diskutiert. Internationale Metaanalysen berichten positive Behandlungseffekte, beinhalten oft aber auch extramurale Settings [4–6] bzw. Nachsorge [7]. Für gefängnisbasierte Behandlungen zeigen sich teils günstige [5, 6], teils ausbleibende [4] oder sogar negative Effekte [8].

Im Jahr 2024 waren in deutschen Gefängnissen 4087 Männer aufgrund eines Sexualdelikts inhaftiert, was einem Anteil von rund 10 % aller Inhaftierten entspricht [9]. Die häufigsten Indexdelikte waren sexueller Kindesmissbrauch (1807 Männer), gefolgt von Vergewaltigung (615 Männer) und Straftaten im Zusammenhang mit Abbildungen sexueller Gewalt an Kindern (459 Personen). Ein Teil dieser Gruppe ist in den 71 sozialtherapeutischen Anstalten (SothA) untergebracht. Dort wurden im Jahr 2024 insgesamt 1900 Personen behandelt, mehr als die Hälfte davon wegen eines Sexualdelikts (53,1 %; [10]). Seit einer Gesetzesnovellierung im Jahr 1998 ist eine sozialtherapeutische Behandlung für Personen, die wegen eines Sexualdelikts zu einer Freiheitsstrafe von mindestens 2 Jahren verurteilt wurden, verpflichtend (vgl. § 9 I StVollzG [11]).

### Rehabilitationstheorien

#### Risk-Need-Responsivity-Modell.

Anfang der 1990er-Jahre wurde das Risk-Need-Responsivity-Modell (RNR) von Andrews et al. [12] konzipiert. Es stellt einen empirisch fundierten und weitverbreiteten Bezugsrahmen für die Behandlungsplanung bei Personen mit Sexualdelinquenz dar. Gemäß *Risk-Prinzip *sollen Umfang und Intensität einer Intervention dem Rückfallrisiko entsprechen, da Straftäter mit moderatem bis hohem Risiko besonders von der Behandlung profitieren, während bei Niedrigrisikogruppen übermäßige Behandlung sogar kontraproduktiv sein kann [13]. Das *Need-Prinzip* konkretisiert, dass sich die Behandlung auf die Veränderung dynamischer, kriminogener Risikofaktoren richten soll, da diese mit dem Rückfallrisiko verknüpft sind. Das *Responsivity-Prinzip* betont die Notwendigkeit der Passung zwischen Methode und Behandeltem. Dabei sollen gemäß allgemeiner *Responsivity* evidenzbasierte Verfahren angewandt werden, während spezifische Responsivity besagt, dass Interventionen an individuelle Voraussetzungen des Behandelten, wie z. B. Motivation, intellektuelle Fähigkeiten sowie kulturelle Besonderheiten, angepasst werden sollen. Die Adhärenz zu den Prinzipien ist mit verbesserten Behandlungseffekten assoziiert [6, 14], gleichwohl ist das Modell in jüngerer Zeit auch Gegenstand kritischer Diskussion geworden [15].

#### Good Lives Model.

Das Good Lives Model (GLM) betrachtet Straftaten als fehlgeleitete Strategie, grundlegende menschliche Bedürfnisse – etwa Autonomie, Verbundenheit oder Kompetenz, sog. primäre Güter – zu befriedigen. Ziel der Behandlung ist es daher, delinquent gewordene Personen mit internen Fähigkeiten (z. B. Emotions- und Selbstregulation) und externen Ressourcen (Arbeit, stabile Beziehungen) auszustatten, um diese „Güter“ auf legale Weise verwirklichen zu können, sodass delinquente Strategien überflüssig werden [16]. Damit ergänzt das GLM die risikoorientierte RNR-Logik um eine positive, ressourcenbasierte Perspektive [17]. Die Evidenz hinsichtlich rückfallreduzierender Effekte durch Anwendung des GLM ist jedoch gering, gleichwohl bestehen Hinweise auf Verbesserungen in der Therapieadhärenz durch einen stärkenorientierten Ansatz [18, 19].

### Behandlungsansätze

#### Sozialtherapie.

Die Sozialtherapeutischen Anstalten (SothAn) haben sich auf gemeinsame Mindestanforderungen zur Behandlung und Struktur verständigt und verfolgen ein integratives Konzept mit therapeutischer Gemeinschaft und multiprofessionellen Angeboten [20]. Es erfolgen zunächst eine diagnostische Indikationsprüfung und Zielfestlegung [21]. Nach einer Orientierungs- bzw. Probephase beginnt die Behandlungsphase, in welcher an den Behandlungszielen gearbeitet wird, wobei einzel- und gruppentherapeutische Maßnahmen zur Anwendung kommen. Rückverlegungen in den Regelvollzug können bei Disziplinarverstößen (z. B. Substanzkonsum, Tätlichkeiten, Verstößen gegen die Hausordnung) erfolgen. Die Quote (anstaltsseitiger) Therapieabbrüche lag im Jahr 2024 bei 42,7 % [10].

#### Psychotherapieschulen.

In der Psychotherapie von Personen, die ein Sexualdelikt begangen haben, haben sich 3 Ansätze etabliert:

*Kognitiv-verhaltenstherapeutische Maßnahmen* gelten derzeit als der am besten empirisch untersuchte Ansatz in der Behandlung sexualdelinquent gewordener Personen [22, 23]. Im Fokus steht dabei die Veränderung kriminogener Einstellungen und Kognitionen, der Selbstregulationsfähigkeiten, von Intimitätsdefiziten und Beeinträchtigungen der Sexualität, wobei z. B. mittels kognitiver Umstrukturierung, Skills-Trainings oder schematherapeutischer Interventionen verhaltensbezogene, kognitive und emotionale Fähigkeiten gefördert werden [24].

*Psychodynamische Ansätze* sind bisher in der Anwendung bei der betreffenden Gruppe kaum systematisch erforscht. Allerdings liegen inzwischen Ansätze für die Anwendung im forensischen Setting in Form der übertragungsfokussierten Psychotherapie [25] sowie der mentalisierungsbasierten Therapie bei Antisozialität [26] vor, wobei hier erste Hinweise auf Anwendbarkeit auch im intramuralen Kontext vorliegen [27]. Grundlegende Annahmen sind hierbei, dass delinquentes Verhalten als Ausdruck von unbewussten Konflikten, Beziehungsstörungen und/oder strukturellen Defiziten in der Persönlichkeitsorganisation bzw. der Mentalisierungsfähigkeit betrachtet werden kann.

Was *systemische Therapieansätze* anbelangt, die Straffälligkeit multiperspektivisch unter Einbezug des sozialen Systems begreifen, liegen erste vielversprechende internationale Studien für die Anwendung bei sexualdelinquent gewordenen Jugendlichen vor [28, 29], Ergebnisse zu Erwachsenen stehen jedoch noch aus.

Aktuelle Daten in deutschen Anstalten zeigen eine heterogene Versorgungspraxis [30]. Am häufigsten kamen verhaltenstherapeutische Verfahren zum Einsatz (*n* = 67), gefolgt von tiefenpsychologisch fundierten (*n* = 40) und systemischen Ansätzen (*n* = 39). Psychoanalytische Verfahren spielten mit 5 Nennungen nur eine marginale Rolle. Hinsichtlich der Qualifikation verfügten 36,9 % des psychologischen Fachpersonals über eine Approbation, weitere 25 % befanden sich in Ausbildung.

#### Behandlungsprogramme.

Aktuelle bundesweite Daten zur Verbreitung manualisierter Programme zur Behandlung von Menschen mit Sexualdelinquenz in der Vorgeschichte fehlen. Gemäß einer Erhebung aus dem Jahr 2009 [31] wurde das „Behandlungsprogramm für Sexualstraftäter“ (BPS; [32]) am häufigsten angewandt, gefolgt vom „Sexual Offender Treatment Programme“ (SOTP; [33]), dem „Eldridge-&-Bullens-Programm“ [34] und dem „Reasoning-&-Rehabilitation-Programm“ (R&R; [35]) – Letzteres ist für antisoziales Verhalten im allgemeinen konzipiert, wird jedoch auch bei sexualdelinquent gewordenen Personen angewandt.

### Inhalte des Artikels

Ziel dieses Beitrags ist es, den Forschungsstand zur Wirksamkeit intramuraler psychosozialer Behandlungsansätze bei Sexualdelinquenz in deutschen Haftkontexten darzustellen und methodenkritisch zu bewerten. Zunächst werden zentrale theoretische Modelle sowie psychotherapeutische und sozialtherapeutische Behandlungsansätze im Strafvollzug dargestellt. Anschließend werden, differenziert nach Outcome, zunächst Rückfalluntersuchungen und anschließend Studien zu proximalen Veränderungsmaßen präsentiert. Schließlich werden die Hauptergebnisse diskutiert, methodische Herausforderungen aufgezeigt und Schlussfolgerungen für Forschung und Praxis abgeleitet.

## Methode

**Abb. 1:**
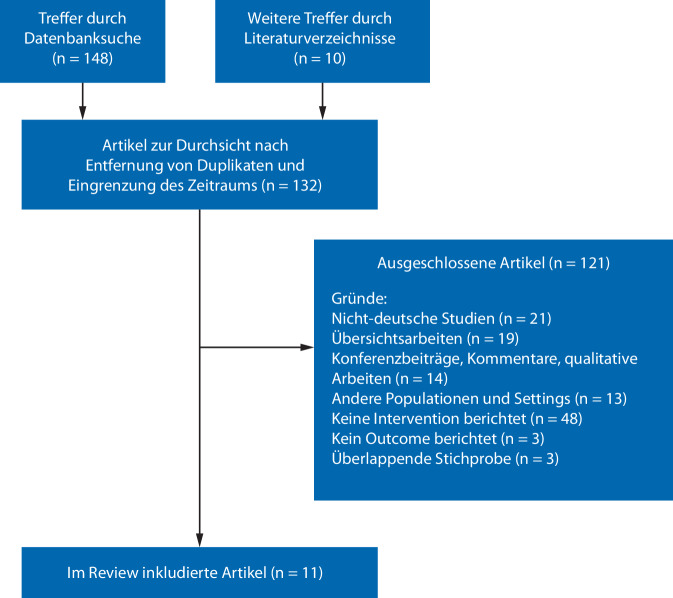
Flowchart der Artikelselektion

Für die Literaturrecherche zu Behandlungseffekten wurden die Datenbanken MEDLINE, PSYNDEX, PsycINFO, PsycArticles (über Ovid) sowie PubMed (Stand: 19.05.2025) durchsucht. Verwendet wurden Suchbegriffe zu den Themenfeldern „Sexualstraftäter“ (sex offender, sexual offender, sexual delinquency, Sexualdelinquenz, Sexualstraftater, Sexualstraftaten), „Behandlung“ (treatment, therapy, psychotherapy, intervention, Sozialtherapie, social therapy, Sexualstraftäterbehandlung), „Strafvollzug“ (prison, incarcerated, incarceration, prison system, Strafvollzug, Haft) sowie dem geografischen Raum (Germany, German). Eingeschlossen wurden Primärarbeiten, die (a) psychotherapeutische oder sozialtherapeutische Interventionen in deutschen Anstalten bei erwachsenen Männern, die wegen Sexualdelikten inhaftiert wurden, untersuchten und (b) zumindest ein Outcome, das den Behandlungserfolg erfasste (Rückfall oder Prä-Post-Vergleich), berichteten. Ausgeschlossen wurden Fallberichte, theoretische Artikel, Übersichtsarbeiten und Studien mit gemischten Deliktgruppen ohne separate Auswertung für Sexualdelikte. Zusätzlich wurde Literatur über die Literaturverzeichnisse des Literaturkorpus herangezogen, um ein umfassendes Review sicherzustellen. Neben peer-reviewten Studien wurden auch Evaluationsberichte und andere Formen „grauer Literatur“ berücksichtigt, sofern sie nachvollziehbare wissenschaftliche Methoden verwendeten. Insgesamt konnten 11 relevante Publikationen in die Auswertung einbezogen werden (Abb. 1).

## Wirksamkeit der Behandlung

### Studien zu Rückfallraten

6 Studien befassten sich mit der Frage, inwiefern sich eine intramurale Behandlung auf die Legalbewährung (d. h. strafrechtlich relevantes Verhalten nach der Entlassung) auswirkt (Tab. 1).

**Tab. 1:** Studien zur Legalbewährung

Studie	Standort	Stichprobe	Methodische Besonderheiten	Katamnesezeitraum	Ergebnis allg. Delinquenz	Ergebnis einschlägige Delinquenz
Lösel et al. (2023; [38])	Multizentrisch, Bayern	*n* = 1245 Personen mit Sexualdelikt	Quasiexperimentell; Propensity Score Matching	M = 9,3 Jahre; SD = 3,5 Jahre; Range: 37–181 Monate	35,9 % (BG) vs. 41,4 % (KG); *p* = 0,05	6,0 % (BG) vs. 8,0 % (KG); n. s.
Wössner (2021; [37])	Sachsen	*n* = 361 Inhaftierte; davon 53 % mit Sexualdelikt	Quasiexperimentell; kein Matching	3 Jahre	Keine Prozentzahlen (BG/KG) für Teilgruppe der Sexualdelinquenz berichtet	6,0 % (BG) vs. 0,0 % (KG); keine Signifikanzprüfung möglich
Bussmann und Richter (2013*; [41])	JVA Halle, Sachsen-Anhalt	*n* = 380 Inhaftierte; davon 83 % mit Sexualdelikt	Quasiexperimentell; ohne Matching	≥ 2 Jahre; M = 5,5 Jahre	41,8 % (BG) vs. 45,8 % (KG); n. s.	6,7 % (BG) vs. 6,7 % (KG)
Stadtland (2004; [36])	JVA Bützow, Mecklenburg-Vorpommern	*n* = 119 mit Sexualdelikt	Retrospektives Kohortendesign; kein Matching; 2 Kontrollgruppen: Begutachtete (KG1) und Behandlungsabbrecher (KG2)	Median: 9 Jahre; Range: 1–340 Monate	59,9 % (BG) vs. 43,5 % (KG1) vs. 93,3 % (KG2); Signifikanzniveau nicht klar berichtet	30,1 % (BG) vs. 19,6 % (KG1), vs. 40 % (KG2); Signifikanzniveau nicht klar berichtet
Ziethen (2002*; [39])	JVA Berlin-Tegel	*n* = 132 mit Sexualdelikt	Quasiexperimentell; Matching nach Aktenanalyse	≥ 2 Jahre; M = 7,3 Jahre, SD = 2,77	78,8 % (BG) vs. 74,2 % (KG); n. s.	27,3 % (BG), 27,3 % (KG)
Ortmann (2002; [40])	Nordrhein-Westfalen	*n* = 223 Inhaftierte; davon *n* = 49 mit Sexualdelikt	Experimentelles Längsschnittdesign; Randomisierung	5 Jahre	60,0 % (BG) vs. 78,9 % (KG); n. s.	20,0 % (BG) vs. 26,3 % (KG); n. s.

*Unveröffentlichter Forschungsbericht bzw. Diplomarbeit

*BG* Behandlungsgruppe, *JVA* Justizvollzugsanstalt, *KG* Kontrollgruppe, *M* Mittelwert, *n.* *s.* nicht signifikant, *SD* Standardabweichung

#### Studienergebnisse.

Die mittleren Rückfallraten im Hinblick auf allgemeine Delinquenz rangierten in den Behandlungsgruppen zwischen 35,9 % und 78,8 %, für die unbehandelten Kontrollgruppen zwischen 41,4 % und 78,9 %. Bezogen auf einschlägige Rückfalldelinquenz, also die erneute Straffälligkeit im gleichen Deliktbereich, reichten die Raten bei den Behandelten von 6,0 % bis 30,1 %, bei Unbehandelten zwischen 0,0 % bis 27,3 %. Zu beachten ist hier, dass sich die Katamnesezeiträume (Zeiträume der Nachuntersuchung) in den Studien stark unterschieden.

Alle 6 Studien machten Angaben zu Therapieabbrüchen (Drop-outs). Dabei war in 80 % der Studien ein Therapieabbruch mit einem höheren Rückfallrisiko behaftet [36–40], lediglich in einer Untersuchung schnitten Drop-outs sogar geringfügig besser ab [41]. Lösel et al. [38, 42] untersuchten das Problem der Therapieabbrüche im Detail, wobei sich zeigte, dass vor allem Spätabbrecher (nach > 24 Monaten) ein signifikant erhöhtes Rückfallrisiko aufwiesen.

3 Studien untersuchten die Dauer bis zum Rückfall. Lösel et al. [38] zeigten, dass sich allgemeine Rückfälle bei Behandelten im Mittel um knapp 8 Monate verzögerten (36,1 vs. 28,6 Monate). Hinsichtlich einschlägiger Delikte zeigte sich ein diskret gegenläufiger Trend (49,8 vs. 42,7 Monate). Bussmann und Richter [41] fanden bei Sexualdelikten hingegen ein längeres straffreies Intervall (24,8 vs. 16,1 Monate). Bei Ziethen [39] wiesen beide Gruppen ähnliche Rückfalllatenzen von durchschnittlich 14,1 Monaten auf.

3 Untersuchungen betrachteten den Schweregrad der Rückfälle. Lösel et al. [38] zeigten mittels eines Deliktschwereindex, dass es in der Behandlungsgruppe zu einer stärkeren Abnahme der Deliktschwere bei Rückfallereignissen kam. In der Berliner Untersuchung [39] erhielten Behandelte kürzere neue Freiheitsstrafen (im Durchschnitt 19,8 vs. 31,9 Monate). Bei Bussmann und Richter [41] traten zwar gleich häufig Rückfälle mit Sexualdelinquenz auf, doch führten sie bei Behandelten seltener zu erneuten Haftstrafen (14,5 % vs. 24,5 %).

#### Studiencharakteristika.

Lediglich Ortmann [40] arbeitete mit einem randomisierten Design: Zunächst wurden geeignete Therapiebewerber paarweise zusammengeführt, dann wurde die Zuweisung zur Behandlungs- oder Kontrollgruppe per Zufall entschieden. Die restlichen Studien nutzten quasiexperimentelle Designs von unterschiedlicher methodischer Güte. 2 Studien arbeiteten mit unterschiedlich differenzierten *Matching*-Techniken, um die fehlende Randomisierung zu kompensieren. So nutzten Lösel et al. ein Propensity Score Matching, während Ziethen [39] auf Basis von risikoprognostisch relevanten Akteninformationen Paare bildete. 4 Arbeiten basierten hingegen auf natürlichen Vergleichsgruppen, die sich teils erheblich in risikorelevanten Merkmalen unterschieden [36, 37, 41].

Alle Studien griffen zur Erfassung von Rückfällen auf Bundeszentralregister-Daten zurück. Der Range der Katamnesezeiträume reichte von einem Monat bis zu 28 Jahren: Einige Studien nutzten genau festgelegte Zeiträume [37, 40, 43], während 3 Studien Mindestzeiträume angaben [38, 39, 41] und eine Studie einen sehr weit gestreuten Range nannte [36].

Die *Stichprobengrößen* rangierten zwischen sehr kleinen Teilstichproben von 49 Personen bis zu 1245 Probanden. 3 Studien [36, 38, 39] stützten sich auf klar abgegrenzte deliktspezifische Stichproben. In 3 Studien [37, 40, 41] wurde mit gemischten Deliktsstichproben gearbeitet, jedoch wurden hier zumindest Teilergebnisse zu Sexualdelinquenz berichtet, wenngleich sich in einer Studie nur eingeschränkte Angaben zum Teilsample mit Sexualdelikten finden ließen [37]. 80 % der Studien untersuchten einzelne Behandlungsstandorte, während 2 Untersuchungen *multizentrisch* vorgingen [38, 40].

Psychiatrische Diagnosen wurden nur in 3 Untersuchungen erfasst [37, 38, 41], keine von ihnen berichtete indes deren Einfluss auf Behandlungsergebnisse. Risikoprognoseinstrumente (d. h. auf statistischen Modellen beruhende Verfahren zur Rückfallvorhersage) wurden in 3 Studien erwähnt [37, 38, 41], wobei jedoch nur eine Studie [38] diese in Berechnungen einbezog, während sich bei Wössner [37] erhebliche Unterschiede im Basisrisiko zwischen den Vergleichsgruppen (VRAG-Differenz: ca. 4 Punkte) zeigten.

Hinsichtlich der berichteten *Interventionen* benannten alle 6 Studien sozial- bzw. soziotherapeutische Maßnahmen für die Behandlungsgruppe, während eine Studie ebenfalls therapeutische Angebote im Regelvollzug miteinbezog [38]. Details zu Ausmaß und Art der therapeutischen Maßnahmen wurden in 4 Untersuchungen berichtet [38, 40, 41], wobei nur 3 Studien Angaben zu psychotherapeutischen Richtungen machten [38, 39]. Keine der Studien machte Aussagen zu Therapeutenvariablen hinsichtlich Fachkunde und Grad der Ausbildung. 4 Studien verwiesen auf das RNR-Modell [38–41], wobei es in 2 Untersuchungen auch explizit im therapeutischen Konzept benannt wurde [38, 41], eine Studie nannte darüber hinaus das Good Lives Model [38]. Nur eine Studie untersuchte den Zusammenhang zwischen Arten der therapeutischen Interventionen, wie z. B. Wohngruppengesprächen oder Einzeltherapie, und der Legalbewährung [40], berichtete jedoch keine differenzierten Einflüsse auf die Sexualdelinquenz.

Mehrere Studien [38, 39, 41] berichteten von dem methodischen Problem, dass kein echter „No-treatment“-Vergleich möglich war, da sich Hinweise darauf ergaben, dass Teilnehmer der Kontrollgruppe therapeutische Maßnahmen in unklarem Ausmaß bekommen hatten, wobei sich nur 2 Studien darum bemüht hatten, diese Schwierigkeit zu berücksichtigen [38, 39].

### Studien zu proximalen Veränderungen

Eine alternative Möglichkeit, Behandlungseffekte zu erfassen, stellt die Untersuchung von Veränderungen im Verlauf der Behandlung dar. Dabei wird die Auswertung proximaler Kriterien wie Veränderungen in empirisch fundierten Risikofaktoren vorgenommen. Es konnten 5 Studien identifiziert werden (Tab. 2).

**Tab. 2:** Studien zu proximalen Behandlungseffekten

Studie	Standort	Stichprobe	Design/methodische Besonderheiten	Outcome (proximale Veränderung)	Ergebnis
Schwedler und Wößner (2013; [44])	Sachsen	*n* = 153; *n* = 86 mit Sexualdelikt	Quasiexperimentell; keine Randomisierung, kein Matching; 2 Messzeitpunkte	Selbstkontrolle, Aggressivität und Erregbarkeit, Persönlichkeitsfaktoren; Empathie; kriminogene Einstellung (nur Selbstbericht)	Signifikanter Behandlungseffekt der SothA nur bei emotionaler Labilität ↓, sonst keine Gruppenunterschiede
					Prosoziale Veränderungen bei Personen mit Sexualdelikt eher im Regelvollzug als in SothA
Dahle et al. (2018; [45])	Berlin	*n* = 105 mit Sexualdelikt	Quasiexperimentell; naturalistisches 3‑Gruppen-Design (Therapie/Warteliste/Freigänger); multimethodisch; 4 Messzeitpunkte	Dynamische Risiko- und Schutzfaktoren, Selbst- und Fremdbericht; Regelverstöße	↓ dynamisches Risiko
					LSI-R: d = 0,73
					HCR-20-C: d = 0,96
					HCR-20-R: d = 1,57
					STABLE-2007: d = 1,68
					Geringere Veränderungen bei TN in Warteliste und vor Beginn der Behandlung
Hosser und Weber (2021; [46])	Multizentrisch; Mecklenburg-Vorpommern und Niedersachsen	*n* = 134; *n* ≈ 16 mit Sexualdelikt	Quasiexperimentell; Kontrollgruppe; retrospektives Matching auf Aktenbasis; 3 Messzeitpunkte	Aggression, Impulsivität, Normorientierung, Selbstwert, Empathie; Behandlungsmotivation; Regelverstöße	Positive, kleine bis mittlere Effekte (d: 0,23–0,48); Effekte stabil auch nach 3 Monaten; Regelverstöße ↓ bei BG vs. ↑ bei KG
Stück, Briken und Brunner (2022; [48])	Hamburg	*n* = 146 mit Sexualdelikt	Reiner Prä-Post-Vergleich; 2 Messzeitpunkte; keine Kontrollgruppe	Dynamische Risikofaktoren (Stable-2007)	↓ Dynamisches Risiko
					Mittlere Differenz Stable-2007 = −0,39 (SD = 1,8; n. s.)
Wischka (2013; [47])	Niedersachsen, multizentrisch	*n* = 320 mit Sexualdelikt	Reiner Prä-Post-Vergleich; 2 Messzeitpunkte; keine Kontrollgruppe	Persönlichkeitsfaktoren, deliktrelevante Einstellungen, Opferempathie	Prä-Post-Veränderungen
					↓ deliktrelevanter Einstellungen
					↑ protektiver Persönlichkeitsfaktoren
					↑Opferempathie

*BG* Behandlungsgruppe, *HCR-20* Historical, Clinical, Risk Management (Risikobewertungsinstrument für Gewalttaten), *HCR-20‑C* Clinical Scale, *HCR-20‑R* Risk-Scale, *KG* Kontrollgruppe, *LSI‑R* Level of Service Inventory – Revised (Risikobewertungsinstrument für allgemeine Delinquenz), *SD* Standardabweichung, *SothA* Sozialtherapeutische Anstalt, *STABLE-2007* Risikobewertungsinstrument für dynamische Risikofaktoren bei Sexualdelinquenz, *TN* Teilnehmer

#### Studienergebnisse.

Insgesamt berichten die meisten Studien über positive Veränderungen in risikorelevanten Faktoren im Verlauf der Behandlung. Eine Studie [44] fand moderate bis hohe Effekte in Bezug auf das strukturierte Risikoassessment, also die expertengeleitete Einschätzung des Rückfallrisikos mithilfe standardisierter Instrumente. Dynamische (d. h. veränderbare) Risikofaktoren waren nach einer 12-monatigen Behandlung reduziert, insbesondere bei Personen mit höherem Ausgangsrisiko. Die Effekte traten erst nach Behandlungsbeginn, nicht während der Wartezeit in Haft auf. 2 Studien [46, 47] stellten eine Reduktion kriminogener Einstellungen im Selbstbericht fest. Prokriminogene Persönlichkeitsanteile, wie z. B. Impulsivität, Aggression und Anspannung, verbesserten sich im Selbstbericht im Rahmen der Behandlung in 2 Studien [46, 47]. Auch eine Zunahme protektiver Faktoren wurde in 3 Untersuchungen berichtet, Ressourcen wie (Opfer‑)Empathie und Selbstwert verbesserten sich im Laufe der Behandlung [46, 47]. Eine Studie [48] fand jedoch, dass Inhaftierte mit Sexualdelikten im Regelvollzug ohne sozialtherapeutische Behandlung günstigere Veränderungen in kriminogen relevanten Merkmalen aufwiesen. Hinsichtlich des Einflusses der Behandlung auf Regelverstöße bleibt das Bild unklar, eine Untersuchung stellte eine 75 %-ige Reduktion disziplinarischer Auffälligkeiten im Nachgang einer Behandlung fest [44], in einer anderen [46] wies hingegen die Behandlungsgruppe mehr Regelverstöße auf als die Kontrollgruppe.

#### Studiencharakteristika.

Nur 3 Studien nutzten quasiexperimentelle Designs mit Kontrollgruppen. Dabei kontrollierte die Untersuchung von Dahle et al. [44], die eine „natürliche“ Warteliste nutzte, am meisten Störeinflüsse. Eine Studie kompensierte fehlende Randomisierung durch ein Matched-pairs-Verfahren [46], während die dritte quasiexperimentelle Studie [48] keine Parallelisierung der Gruppen vornahm.

Die Hälfte der Studien arbeitete mit 2 Messzeitpunkten [45, 47, 48], während eine Studie eine Follow-up-Messung 3 Monate nach Beendigung der Intervention vornahm [46]. Eine weitere Arbeit nutzte 4 Messzeitpunkte, allerdings teils retrospektiv [44]. Als methodische Einschränkung ist bei Schwedler und Wössner [48] zu beachten, dass der Zeitraum zwischen den Messzeitpunkten in der Behandlungsgruppe nahezu doppelt so lang war wie in der Kontrollgruppe. Keine der Untersuchungen umfasste einen Katamnesezeitraum, der über die Haftzeit hinausging.

Alle 5 Studien nannten sozialtherapeutische Behandlungskonzepte. In 3 Studien wurden Rehabilitationsmodelle, vorrangig das RNR-Modell, genannt [44, 45, 47], eine Studie erwähnte das Good Lives Model [44]. Detaillierte Angaben zu Therapierichtungen und -inhalten wurden nur in 2 Studien genannt, die explizit ein Behandlungsprogramm prüften [46, 47]. Jedoch nutzte nur eine dieser Untersuchungen eine Kontrollgruppe [46], allerdings war der Anteil an Menschen mit Sexualdelikt in beiden Gruppen sehr gering (11,7 % bzw. 9,5 %) und das Programm nicht spezifisch auf diese Deliktgruppe ausgelegt. Informationen zur fachlichen Qualifikation bzw. Fachkunde der Therapeut:innen wurden in keiner Studie berichtet. Psychiatrische Diagnosen wurden nur in 2 der Arbeiten erwähnt [44, 45].

Alle 5 Studien gaben an, Risiko- und Schutzfaktoren zu erfassen, wobei diese sehr unterschiedlich erfasst wurden. So nutzte eine Studie ausschließlich Selbstberichtsskalen [48], während 2 Studien [44, 46] neben Selbstberichtsdaten als externes Kriterium Regelverstöße einbezogen. Nur 2 Untersuchungen nutzten Risikoprognoseinstrumente im Prä-Post-Vergleich [44, 45]. Wischka [47] bezog neben Selbstberichtsdaten ein Fremdrating der Opferempathie anhand qualitativer Auswertung der Opferbriefe ein.

## Diskussion

Die vorliegende Übersicht berichtet über 11 Arbeiten in der deutschen Versorgungslandschaft zur intramuralen Behandlung von Männern, die Sexualdelikte begangen haben. Die methodische Qualität und die Evaluationsergebnisse der ausgewerteten Arbeiten sind sehr heterogen: Hinsichtlich des Effekts auf die Legalbewährung ist die Evidenz inkonsistent. Einige Studien berichten zwar günstigere allgemeine Rückfallraten [38, 40, 41], signifikante Unterschiede ergeben sich jedoch nur in der größten Stichprobe [38]. Die Effektstärken waren auch bei den Untersuchungen, die positive Behandlungsergebnisse berichteten, klein [49]. Bei einschlägigen Rückfällen ergibt sich ein noch uneinheitlicheres Bild: teils niedrigere [38, 40], teils keine [39, 41] oder sogar höhere Raten [37]. Ein ähnlicher Befund ergibt sich auch mit Blick auf die internationale Diskussion zur Wirksamkeit von sexualforensischen Psychotherapien [50]. Auch hinsichtlich differenzierterer Rückfallkriterien sind die Ergebnisse uneinheitlich. Wenngleich Behandelte tendenziell später und mit weniger schwerwiegenden Delikten rückfällig wurden, wiesen sie dennoch relevante Rückfallraten auf.

Nahezu einhellig zeigten die Studien, dass ein Abbruch der Behandlung mit höheren Rückfallrisiken verbunden war. Auch dies stimmt mit Forschungsbefunden aus anderen Ländern überein [51]. Ob dies an destabilisierenden Effekten des Abbruchs selbst, an Charakteristika der Abbrechergruppe oder aber an der fehlenden therapeutischen Berücksichtigung von Besonderheiten dieser Gruppe liegt, bleibt offen, hier bedarf es weiterer Forschung [52] und möglicherweise responsivitätsorientierter Anpassungen, um Abbrüchen entgegenzuwirken. Die zunehmende Quote an anstaltsseitigen Erledigungen der Sozialtherapie stimmt bedenklich [10], da gerade jene ein höheres Risiko und gemäß RNR-Logik einen höheren Behandlungsbedarf aufweisen.

Es konnten insgesamt nur sehr wenige Studien identifiziert werden. Ein Grund dafür liegt in der erschwerten Durchführung randomisiert kontrollierter Studien im Strafvollzug, da ethische Bedenken hinsichtlich der Freiwilligkeit der Mitwirkung bei Inhaftierten bestehen und eine Zuweisung zu unbehandelten Kontrollgruppen eine Ungleichbehandlung darstellen könnte. Zudem bestehen datenschutzrechtliche Hürden. Dennoch zeigt die Studie von Ortmann [40], dass kontrollierte Studien auch in sozialtherapeutischen Anstalten möglich sind. Hinsichtlich der Kontrolle von Selektionseffekten überrascht es kaum, dass Studien, die eine High-Risk-Behandlungsgruppe mit einer Kontrollgruppe mit weniger schwerwiegenden Delikten verglichen, keinen Vorteil für die Behandelten finden. Die Bedeutung der Bildung risikoäquivalenter Vergleichsgruppen bei künftigen nicht randomisiert kontrollierten Untersuchungen wird ferner dadurch unterstrichen, dass bei Anwendung des RNR-Prinzips definitionsgemäß Selektionseffekte auftreten, insofern hiernach insbesondere jene mit hohem Rückfallrisiko behandelt werden sollten.

Die teilweise kleinen (Teil‑)Stichprobengrößen stellen insbesondere vor dem Hintergrund des Problems der geringen Basisraten für einschlägige Rückfälle ein methodisches Problem dar. Die Wiederverurteilungsrate für Sexualdelikte im deutschen Sprachraum liegt im einstelligen Prozentbereich [2, 3], was weitestgehend mit den hier berichteten Ergebnissen korrespondiert – wobei sich ein Trend zeigte, dass neuere Studien tendenziell niedrigere Rückfallraten berichteten. Somit lassen sich bei den erwartbaren niedrigen oder allenfalls moderaten Effektstärken nur in großen, risikovergleichbar zusammengesetzten Gruppen signifikante Effekte erwarten. Die Ergebnisse der einzigen randomisierten Untersuchung [40] sind aufgrund der geringen (Teil‑)Stichprobengröße nur eingeschränkt verwertbar. Doch selbst in der größten Studie [38], bei der Behandelte ein 25 % geringeres Risiko aufwiesen, einschlägig rückfällig zu werden, illustrieren die absoluten Rückfallzahlen, dass hier bereits einzelne Rückfälle ausreichen würden, um die Ergebnisse zu verändern.

Demgegenüber liefern die proximalen Effekte der Behandlung ein homogeneres und überwiegend positives Ergebnis, weisen jedoch methodische Mängel auf. Nur die Hälfte nutzte überhaupt Kontrollgruppen. Es ging kein Katamnesezeitraum über den Haftzeitraum hinaus. Alle berücksichtigten Studien untersuchten zwar sogenannte Risikofaktoren als Outcomes, deren Operationalisierung war jedoch teils problematisch. So ist die Validität von Selbstberichtsverfahren bei forensischen Populationen umstritten [53], da soziale Erwünschtheit, aber auch Hoffnung auf institutionelle Begünstigung eine Rolle spielen können. Externe Maße, wie Disziplinarverstöße, sind nur in Teilen objektiv, insofern sie z. B. vom anstaltsspezifischen Anzeigeverhalten abhängig sind. Fremdeinschätzungen der Opferempathie [47] erscheinen fehleranfällig und nur unzureichend empirisch fundiert. Positiv hervorzuheben ist die Untersuchung von Dahle [44], die sowohl mehrere Perspektiven berücksichtigte – inklusive aktuarischer Risikoprognoseinstrumente – und zudem mit einer natürlichen Wartegruppe operierte. Allerdings untersuchte keine der Arbeiten mit sexualdelinquent gewordenen Personen, inwiefern sich Veränderungen in risikorelevanten Merkmalen auf die Legalbewährung auswirkten.

Ein Forschungshindernis liegt darin, dass diagnostische Ergebnisse nicht ausreichend berichtet wurden. Psychiatrische Diagnosen wurden in einigen Studien zwar erwähnt, jedoch nicht systematisch erfasst oder berichtet oder in Bezug zu Behandlungsergebnissen gesetzt. Häufig wurden Grundsätze des RNR-Modells benannt, es blieb jedoch unklar, inwiefern diese in der Behandlung umgesetzt wurden. Lediglich 4 Studien berichteten über den Einsatz aktuarischer Risikoprognoseinstrumente.

Weitere Defizite wies die Studienlage hinsichtlich Aspekten auf, die die Behandlungsintegrität betreffen. So fehlten weitestgehend Angaben zu Behandlungsprogrammen und -durchführung, während die restlichen Studien zwar größtenteils eine sozialtherapeutische Behandlung berichteten, deren Art, Umfang und Inhalte jedoch nicht weiter spezifizierten. Lediglich eine Studie berichtete differenzielle Ergebnisse zu sozialtherapeutisch und im Regelvollzug Behandelten [38], wobei der Befund, dass beide Gruppen im Vergleich zur unbehandelten Vergleichsgruppe profitierten, dafür spricht, dass Faktoren der Behandlung als solche – und nicht etwa unspezifische haftassoziierte Faktoren – eine Auswirkung haben. Es bleibt unklar, welche Komponenten der Behandlung für die Effekte verantwortlich sind. Wiederum analog zur Diskussion der internationalen Studienlage könnte hierfür z. B. die strenge Berücksichtigung der RNR-Prinzipien [14] oder auch unspezifische Wirkfaktoren von Psychotherapie [54] bedeutsam sein. Auch bestehen Schwierigkeiten, eine eindeutige No-treatment-Bedingung in der Kontrollgruppe zu gewährleisten, da nicht sicher erfasst werden konnte, inwiefern Kontrollprobanden Behandlungsangebote bekommen hatten.

Keine Arbeit untersuchte Zusammenhänge zwischen Behandelnden-Variablen – wie Approbation, Fachkunde, Haltung – und Behandlungsoutcomes. Dabei ist bekannt, dass sich Effekte der Behandlung insbesondere dann zeigen, wenn sie von fachlich qualifizierten Behandelnden angeboten wird [6, 23]. Aus der Stichtagserhebung [30] lässt sich schließen, dass die personellen und konzeptionellen Rahmenbedingungen in den SothAn uneinheitlich sind und nur 37 % des Fachpersonals über eine Approbation verfügen, wenngleich die Mindeststandards dies empfehlen. In der Behandlungspraxis zeigt sich ein therapeutischer Eklektizismus, der im Gegensatz zur ambulanten Regelversorgung nicht durch Gutachterverfahren reguliert wird. Verhaltenstherapeutische Ansätze sind bei Sexualdelinquenz zwar in internationalen Studien gut erforscht [22], Überprüfungen im deutschen Strafvollzug fehlen jedoch. Zugleich zeigte sich, dass tiefenpsychologisch fundierte Verfahren zu den am zweithäufigsten eingesetzten Therapieansätzen zählen, wobei auch deren Evaluation im deutschen Sprachraum bislang nicht erfolgt ist. Diese Versorgungsrealität spiegelt sich im deutschsprachigen Forschungsdiskurs bislang kaum wider.

### Limitationen der vorliegenden Arbeit.

Es erfolgte lediglich eine narrative Einordnung ohne Gewichtung der Studien, wie es z. B. anhand der Maryland Scientific Methods Scale möglich wäre. Zudem konnten insgesamt nur wenige Studien in die Analyse aufgenommen werden. Das zusätzliche Heranziehen von Studien über Literaturverzeichnisse, auch grauer Literatur, also Arbeiten, die ohne Peer-Review-Verfahren publiziert wurden, erweitert zwar die Datenbasis, schränkt jedoch aufgrund fehlender Qualitätskontrolle die Belastbarkeit der Ergebnisse ein.

## Fazit und Ausblick

Insgesamt fiel ein Informationsmangel in Bezug auf die intramurale Versorgungssituation in Deutschland auf. Auf Basis des aktuellen Forschungsstands ist nur eine sehr vorläufige Bewertung der Wirksamkeit intramuraler Behandlungsformen bei Sexualdelinquenz möglich. Die teils verhaltenen Ergebnisse lassen sich jedoch nicht sicher als Versagen therapeutischer Maßnahmen interpretieren, vielmehr müssen sie vor dem Hintergrund methodischer Herausforderungen betrachtet werden. Aktuell erscheint die Forderung des Strafvollzuggesetzes nach einer Forschungsbegleitung der Behandlung in den Haftanstalten nicht ausreichend erfüllt.

Es bedarf weiterer breit angelegter, methodisch hochwertiger Studien mit Kontrollgruppen, um Behandlungseffekte abzubilden. Um randomisiert kontrollierte Studien zu ermöglichen, bedarf es der Unterstützung durch die Justizministerien bzw. -behörden. Alternativ muss fehlende Randomisierung ggf. mittels elaborierter Matching-Techniken kompensiert werden, wobei unbedingt für das Risikolevel kontrolliert werden muss, um inhaltliche Aussagen zu Behandlungseffekten zu ermöglichen. Um statistisch signifikante Ergebnisse zu erzielen, muss dem Problem der niedrigen Basisraten bei einschlägigen Rückfällen begegnet werden.

Hierzu bieten sich mehrere Strategien an: Die Stichproben sollten groß sein, wobei multizentrische, ggf. auch bundesweite Kooperationen sinnvoll wären, um Effekte zu detektieren. Hier sollten auch die kriminologischen Dienste der Länder einbezogen werden, um dem gesetzlich verankerten Forschungsauftrag nachzukommen. Ferner könnte ein Rückgriff auf sensitivere Rückfallmaße, wie polizeiliche Anzeigen (z. B. MESTA- oder POLIKS-Daten) hilfreich sein, wie auch die Nutzung längerer Katamnesezeiträume, um Effekte zu identifizieren. Auch die Integration von Evaluationsbemühungen in die tägliche Arbeit der Behandelnden, z. B. durch eine standardisierte Diagnostik unter Berücksichtigung der RNR-Prinzipien, aktuarischer Risikoinstrumente sowie psychiatrischer Diagnosen, sowie einheitliche Behandlungsstandards bzw. die Erfassung von Therapieausmaß und -art erscheinen sinnvoll. Dies kann auch die weniger aufwendigen Untersuchungen zu proximalen Veränderungen befruchten.

Eine Implementierung systematischer Erhebungsmethoden im Regelvollzug erscheint notwendig, da die dortigen Behandlungsmaßnahmen bisher zu wenig erforscht erscheinen. Proximale Veränderungen wie auch Rückfälle können als Teile einer Ergänzungsreihe betrachtet werden, da sie potenziell aufeinander bezogene Aspekte desselben Veränderungsprozesses darstellen. Die Analyse ihrer Zusammenhänge kann Hinweise darauf geben, welche therapeutischen Veränderungen für eine nachhaltige Legalbewährung besonders bedeutsam sind. Hierfür erscheinen eine enge Kooperation und Kommunikation von Forschung, Justiz und Strafvollzug sinnvoll.
